# Tailored CNTs Buckypaper Membranes for the Removal of Humic Acid and Separation of Oil-In-Water Emulsions

**DOI:** 10.3390/membranes10050097

**Published:** 2020-05-12

**Authors:** Eman Elnabawy, Ibrahim M. A. Elsherbiny, Ahmed M. A. Abdelsamad, Badawi Anis, Abdelwahab Hassan, Mathias Ulbricht, Ahmed S. G. Khalil

**Affiliations:** 1 Physics Department and Center for Environmental and Smart Technology, Faculty of Science, Fayoum University, Fayoum 63514, Egypt; eman.elnabawy@smartci.alexu.edu.eg (E.E.); aha08@fayoum.edu.eg (A.H.); 2 Lehrstuhlfür Technische Chemie II, and Center for Water and Environmental Research (ZWU), University of Duisburg-Essen, 45141 Essen, Germany; ibrahim.elsherbiny@uni-due.de (I.M.A.E.); ahmed.abdelsamad@stud.uni-due.de (A.M.A.A.); mathias.ulbricht@uni-due.de (M.U.); 3 Water Pollution Dept, National Research Centre, 33 El-Bohouth St., Dokki, Giza 12622, Egypt; 4 Spectroscopy Dept, Physics Division, National Research Centre, 33 El-Bohouth St., Dokki, Giza 12622, Egypt; badawi.ali@gmail.com; 5 Materials Science & Engineering Department, School of Innovative Design Engineering, Egypt-Japan University of Science and Technology (E-JUST), 179 New Borg El-Arab City, Alexandria 21934, Egypt

**Keywords:** carbon nanotubes, buckypapers, surface modification, humic acid, oil-in-water emulsions

## Abstract

Carbon nanotubes (CNTs) are a robust material and proven as a promising candidate for a wide range of electronic, optoelectronic and environmental applications. In this work, two different methods were utilized for the preparation of CNTs exhibiting different aspect ratios via chemical vapor deposition (CVD). The as-prepared CNTs were analyzed using scanning electron microscopy (SEM), transmission electron microscopy (TEM), N_2_adsorption isotherms, thermogravimetric analysis and Raman spectroscopy in order to investigate their morphological and structural properties. Free-standing CNTs “buckypaper” membranes were fabricated, characterized and tailored to meet the requirements of two applications, i.e., (1) the removal of humic acid (HA) from water and (2) separation of oil-in-water emulsions. It was revealed that the hydrophobic buckypapers showed high separation performance for Shell oil-in-water emulsions filtration, with up to 98% through the accumulation of oil droplets onto the membrane surface. The absorption capacity of buckypaper membranes for various organic liquids (oil, chloroform and toluene) was evaluated over 10 absorption cycles to investigate their recyclability and robustness. Moreover, surface modification was introduced to the pristine CNTs to increase their surface hydrophilicity and improve the pure water permeability of buckypapers. These modified buckypapers showed high flux for HA solutions and excellent HA rejection efficiency up to 95%via size exclusion and electrostatic repulsion mechanisms.

## 1. Introduction

In the last few years, carbon-based nanomaterials such as carbon nanotubes (CNTs), have received significant interest for wastewater treatment and desalination applications [1,2,3,4,5], owing to their high aspect ratio [6,7], large surface area [8] and ease of functionalization [9,10,11]. CNTs membranes, including free standing or mixed matrix types, have shown a great potential in breaking the trade-off between membrane flux and selectivity in addition to anti-fouling property [12,13,14]. CNTs membranes with “buckypaper” like structure have gained specific attention for their unique advantages, including high porosity and interconnected pore structure [15].

Natural organic matter (NOM) in the aquatic environment consists of a wide variety of organic compounds that are primarily derived from the decomposition of plant and animal residues [16,17]. The presence of NOM traces in water resources is a major concern for environmental scientists [18], specifically in water treatment. Among various hydrophobic NOM, HA shows the greatest potential for forming toxic disinfection byproducts (DBPs) and inhibiting the coagulation efficiencies of many pollutants [19,20]. Moreover, a high concentration of HA in drinking water can also cause a significant increase in color and odor as well as bacterial re-growth. CNTs membranes showed superior performance for the removal of natural organic matters (NOM) [21,22,23] and separation of oil-in-water emulsions compared to conventional polymeric membranes.

Vertically aligned CNTs membrane (VACNTs) with a contact angle of 85° and an average pore size of 20 nm was introduced for the removal of HA from water through dead-end and cross flow filtration [24]. The results showed a remarkable water flux of 3600 ± 100 L·m^−2^h^−1^ at 1 bar, while a rapid HA flux decline of 50% was observed during the first 10 min of filtration. The noticeable membrane fouling was attributed to the adsorption of HA onto the membrane surface and blocking the pores. Similarly, enhanced permeability, but also in combination with fouling resistance toward organic substances, was achieved through CNTs-hollow fiber membrane with electrochemical assistance [25]. The proposed system showed a great potential in the removal of bovine serum, glucose, phenol, bovine serum albumin (BSA), and HA through various mechanisms including size exclusion, electrostatic repulsion and electrochemical oxidation. The CNTs-hollow fiber membrane showed pure water flux of 1250 L·m^−2^h^−1^ at Transmembrane pressure (TMP) of 0.6 bar and electrochemical potential of 1.0 V.

Functionalization of CNTs either by covalent or non-covalent bonding has been developed to enhance the CNTs hydrophilicity and dispersibility in polar solvents [26,27]. Functionalized CNTs are more favorable in water treatment applications for their high water permeability and removal efficiencies for various types of water contaminants [28,29]. Carboxyl functionalized multi-wall CNTs (MWCNTs) were incorporated into a polyamide (PA) reverse osmosis (RO) membrane via interfacial polymerization [30]. The synthesized nanocomposite membrane obtained significant antifouling property to BSA and higher chlorine resistance compared to pristine PA membrane, as result of higher surface hydrophilicity and lower surface roughness.

Moreover, detailed comparison between pristine and functionalized CNTs buckypaper membrane for the removal of natural organic matter (NOM) was reported [31]. The resultant functionalized membrane exhibited higher NOM rejection and water permeability than the pristine membrane.

Recently, CNTs buckypaper membranes were introduced for the separation of oil-in-water emulsions [32]. A facile and robust superhydrophobic grafted polystyrene/carbon nanotube hybrid (PS-CNTs) buckypaper was proposed [33]. The PS-CNTs membranes showed superior separation of water-in-oil emulsions with efficiency of 99% and significant water permeance of (5000 L·m^−2^h^−1^ bar^−1^). In addition, the absorption capacity of the composite membrane was investigated and showed high selectivity toward several organic solvents from water with a weight gain of 270 times relative to its initial weight. Another study introduced a superoleophobicpoly(acrylic acid)-modified CNTs membrane, functionalized with catalytic nanoparticle (Pd@Pt), via an interception method [34]. The produced multilayer composite film efficiently decomposed a wide range of organic pollutants and separated the oil-in-water emulsion over 10 filtration cycles. CNTs-MnO_2_ nanorods membrane was synthesized to achieve efficient separation of emulsified oil/water mixtures [35]. The addition of MnO_2_ nanorods assembled 3D hierarchical structure, which was more favorable for water transport, while the emulsified oil droplets were sieved through sub-micron pore size. The membrane exhibited good recyclability with low fouling rate for long-term filtration. Cross-linked CNTs with highly porous microstructural characteristics were also developed for oil-in-water and water-in-oil emulsion separation [36]. The membrane exhibited noticeable hydrophilicity to water in the air whereas it exhibited superoleophobicity to oil underwater. Furthermore, the novel switchable membrane had permeability of 816, 790, and 695 L·m^−2^·h^−1^MPa^−1^ for emulsions of hexane, dodecane, and toluene in water, respectively.

Overall, many studies investigated improving of water flux and selectivity of CNTs membranes. However, only a few of them introduced the effect of CNTs aspect ratio and surface modification on the physical and structural properties of the prepared membranes. Here, we introduce the fabrication of free-standing pristine and surface modified MWCNTs buckypaper membranes using two different methods. CNTs were prepared via chemical vapor deposition (CVD) with different aspect ratios. The as-prepared buckypapers were utilized for the removal of HA from water and the separation of oil-in-water emulsion. The absorption capacity of buckypapers for various organics was also evaluated, along with examining their recyclability and robustness.

## 2. Materials and Methods

### 2.1. Synthesis of CNTs and Buckypapers

Two types of MWCNTs with different aspect ratios were prepared via the chemical vapor deposition (CVD) furnace by changing the process parameters such as catalyst concentration, active carbon gas, operating temperature, reaction time, and gas flow rates. The synthesis parameters used for both methods are listed in Table 1. The CNTs were produced on a solid mixture of Fe as a catalyst and Mo as a promoter deposited on MgO support. The system was purged with H_2_ as a carrier gas for 30 min to achieve complete catalyst reduction. Then, the gas stream was switched on to inject the hydrocarbon precursor gas. Subsequently, the system was cooled down to ambient temperature in an argon atmosphere. Afterward, the as-prepared CNTs were collected and purified for characterization [37]. Surface modification of CNTs was made by dispersing 1 g of the purified CNTs into a mixture of 150:50 mL of H_2_SO_4_ (98%)/HNO_3_ (65%) solution with vigorous stirring for 3 h at room temperature followed by washing and drying [38].

The dispersions used for the buckypapers were prepared through dispersing 150 mg of the purified CNTs into 300 mL of N-methyl-2-pyrrolidone (99%; Merck, Darmstadt, Germany) followed by sonication with the aid of ultrasonic tip (VCX 750, Sonics, Newtown, CT, USA) at 80 W for one hour in ice bath to avoid the effect of sonication heat on damaging the CNTs structure; then the dispersion was centrifuged at 4000 rpm for 30 min to prevent the presence of aggregated bundles in the resultant dispersion. Buckypapers were prepared through transferring the supernatant dispersion into a vacuum filtration unit and filtering through PVDF membrane support (0.22 µm, Millipore, Darmstadt, Germany); the diameter of the resultant buckypapers was 4.7 cm with CNTs mass loading of 8.64 mg/cm^2^. Surface modified buckypapers were prepared by dispersing the functionalized CNTs into aqueous solvent [39], also followed by filtration deposition. The buckypaper could be easily peeled off from the membrane support after it was completely dried on the vacuum filtration system. Then the free standing membrane was dried in vacuum oven at 100 °C overnight.

### 2.2. Morphological and Structural Characterizations of CNTs and Buckypapers

The morphological structure of CNTs was analyzed using transmission electron microscopy (TEM) (Jeol-JEM-1011) operated at 200 kV. CNTs lengths, number of walls, outer and inner diameters were determined using Image-J software. Thermogravimetric analysis of CNTs was performed on a THASS TGA 1000 instrument using 25mg of sample at a heating rate of 20 °C/min under air atmosphere. Raman spectroscopy was performed using an Enwave ProRaman-L instrument at excitation wavelength (532 nm) from a diode laser with power of 2mW and on average 50 scans. The surface area of CNTs was obtained from the N_2_ adsorption isotherms at 77 K, measured and analyzed via the Brunauer–Emmett–Teller (BET, model using a Quanta-chrome Autosorb instrument, Graz, Austria).

Buckypapers pore size distribution was determined with two different methods. Firstly, averaged surface pore diameter was determined with the aid of scanning electron microscopy (SEM) (Sigma 500 VP, ZEISS, Oberkochen, Germany), at an accelerating voltage of 10 kV. Images were analyzed using ImageJ software. The analysis was obtained by image scaling and segmentation of the pores via a threshold technique. The average pore diameter was determined by assuming that all pores are circular [40]. Secondly, pore size distribution was determined through a gas flow/pore dewetting capillary flow porometer system (Porolux 1000, Benelux Scientific, Eke, Belgium). Perfluorinated wetting liquid (Porefil) was used as a test liquid with a surface tension of 16 mN/m and vapor pressure of 399 Pa; it was applied to wet the membrane, while the sample was pressurized at up to 30 bar under N_2_ gas.

Contact angle measurement was conducted using sessile drop and captive bubble methods for pristine and modified buckypapers, respectively using OCA 15 Plus (Dataphysics GmbH, Filderstadt, Germany).

### 2.3. Buckypapers Filtration Performance

Water permeability was recorded for the pristine and surface modified buckypapers as follows. Firstly, to reach a better wettability for buckypapers, the membranes were immersed in isopropanol for 1 h, followed by immersing in water for another hour. Pure water permeability was measured using a dead-end stirred filtration cell (Amicon cell model 8050, Millipore Corp., Darmstadt, Germany) combined with a pressurized feed reservoir. The membranes were compacted at 2 bars for 1 h and then the initial pure water permeability (PWP)was calculated at (TMP) of 0.5 bars for 3 min according to Equation (1):

PWP = m/(ρ × t × A × ΔP),(1)

where (m) is the mass of collected permeate with density (ρ) for time (t) through membrane surface area (A) at transmembrane pressure (ΔP).

The buckypapers were tailored to be used in two different applications according to their surface properties. The pristine buckypapers have a highly hydrophobic nature, superior absorption capacity of organic substances and good recyclability. They were applied for the separation of oil-in-water emulsions and selective absorption for Shell oil and non-polar organic solvents from water. Separation of oil-in-water emulsion was conducted using a vacuum filtration system by applying pressure of 20–30 mbar at the permeate side using a diaphragm pump (Vacuubrand CVC 2 Chemistry Vacuum System, Wertheim, Germany). The subsequent filtration was carried out with a feed obtained through dispersing 5 wt% of Shell oil (HELIX HX3, 20W-50, Shell Oil Company, Houston, TX, US) into deionized water (DI) water and sonicating using an ultrasonic probe for 30 min in ice bath to prevent the effect of sonication heat on the emulsion particle size. Oil-in-water emulsion was transferred into the filtration system and was suction filtered. The oil separation efficiency was analyzed by measuring the total organic carbon (TOC) of feed emulsion and permeate samples. For testing the short and long-term separation performances of the pristine buckypapers, samples of permeate were taken after 1 h and 12 h of emulsion filtration and analyzed with TOC, dynamic light scattering (DLS), using Zetasizer (NanoZw, Malvern, UK) and optical microscopy. The Shell oil-in-water emulsion feed was diluted before particle size analysis. The absorption performance of buckypapers to toluene, chloroform and Shell oil was investigated by dipping pieces of buckypaper membrane (2 × 2 cm^2^). The sorption capacity was quantitatively studied by calculating the weight gain (wt%), which is defined as the weight of absorbed substance relative to the dried membrane. The recyclability and durability of buckypaper membranes were investigated over 10 cycles. After each sorption cycle, the membrane was immersed in ethanol/water mixture (1:1) for 24 h with continuous shaking and then dried.

Surface modified hydrophilic buckypaperswere evaluated for the removal of HA from water. HA (purity 60%, LobaChemie, Mumbai, India) with concentrations of 5 and 10 ppm was used. The membrane antifouling propensity was determined by measuring the permeability decline during filtration of 100 mL of HA solution. The rejection performance of buckypapers was evaluated by measuring the absorbance of HA using the UV-spectrophotometer at 254 nm of the feed and permeates [41], using the following formula:

R(%) = (1 − At/A0) × 100,(2)

where (R) is the rejection, and (At) and (A0) are the absorbance of feed and permeate, respectively.

## 3. Results and Discussion

### 3.1. Characteristics of CNTs

**Figure 1:**
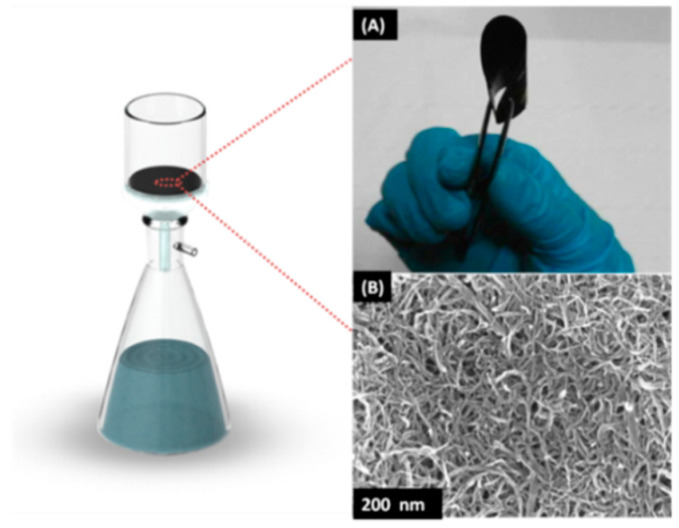
Buckypaper fabrication using vacuum filtration technique: (**A**) as prepared buckypaper with high flexibility and mechanical robustness and (**B**) scanning electron microscopy (SEM) image of the buckypaper surface.

The overall fabrication process of the CNTs buckypaper membrane is illustrated in Figure 1. It can be seen that the optimized flexible buckypaper membrane can berolled and folded without observed cracks as a result of good dispersion stability and homogeneity (Figure S1 in Supplementary Materials).

**Figure 2:**
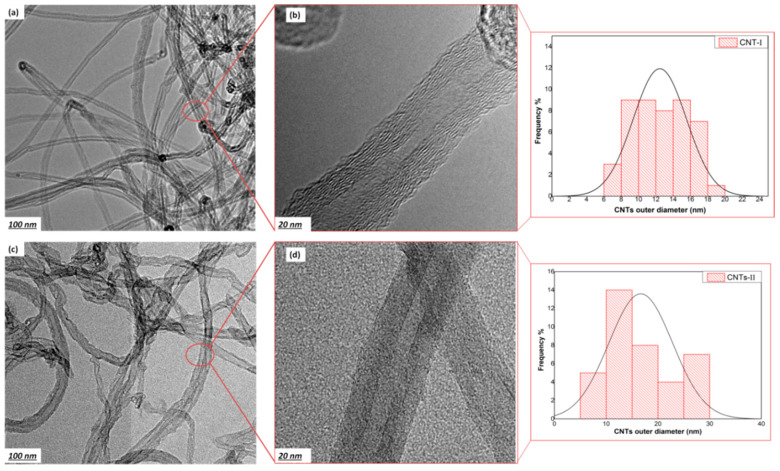
Transmission electron microscopy (TEM) images with low and high magnifications for the two pristine CNTs: (**a**,**b**) CNT-I and (**c**,**d**) CNT-II, combined with outer diameter distribution.

Figure 2 represents the low and high magnifications of TEM images for the two CNTs sources. The CNTs were entangled in the form of agglomerated bundles with no damage on the CNTs edges, while the amorphous carbon and metal catalyst residues were successfully removed by the purification. The TEM images confirm the homogenous structure of CNTs with high quality and integrity [42]. The aspect ratio of CNTs was estimated to investigate the difference between the two preparation methods. CNT-I showed a higher aspect ratio of 284–338 with estimated length of 3.7 ± 0.7 µm as well as outer and inner diameters of 12 nm and 5 nm, respectively, while, CNT-II obtained a calculated aspect ratio of 125–130 with an average length of 2 ± 0.1 µm and about 20 nm and 10 nm outer and inner diameter, respectively. The difference in the aspect ratio can be attributed to the effect of active gas, reaction time and temperature on the growth of CNTs [43]. Li et al. [44] showed that methane is preferred for the growth of smaller diameter and higher purity CNTs compared to other hydrocarbon gases, as it is relatively chemically stable at high temperature.

**Figure 3:**
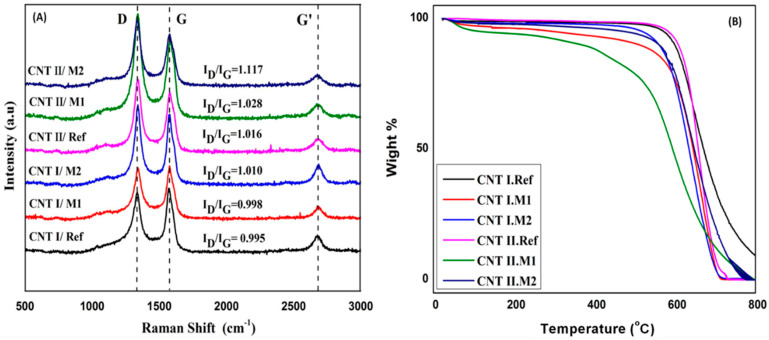
(**A**) Raman spectroscopy and (**B**) thermogravimetric analysis for the pristine and surface modified CNTs.

The quality of CNTs can be further confirmed by Raman analysis. As shown in Figure 3a, two characteristic peaks at ∼1341 and 1570 cm^−1^ were recorded for each sample. The peak at 1341 cm^−1^ is attributed to the D band, which reflects the defects in the CNTs structure, while the peak at 1570 cm^−1^ represents the in-plane vibration of the (C–C) bonds [45]. The relative intensity ratio of these two bands (I_D_/I_G_) was determined to evaluate the degree of defects and functionalization on CNTs. The two CNTs types showed a relatively close I_D_/I_G_ ratio of 0.995 and 1.016 for CNT-I and CNT-II, respectively. Moreover, the surface modified CNTs did not show a noticeable difference in the I_D_/I_G_ ratio for CNT-I and CNT-II. This indicates that no significant damaging on CNTs walls occurred, confirming that the surface modification was successful on the CNTs surface without affecting the tubes internal structure [46].

Thermogravimetric analysis was performed to detect the quality of pristine and surface modified CNTs. Figure 3b represents the CNTs weight loss as a function of temperature up to 800 °C. The decomposition temperature reflects the material’s thermal stability and functionalization degree [47,48,49]. Thus, for the pristine CNTs, constant weight was maintained up to 700 °C, while the surface modified CNTs showed earlier decomposition at 500 °C that can be attributed to the attachment of functional groups onto the CNTs walls.

**Figure 4:**
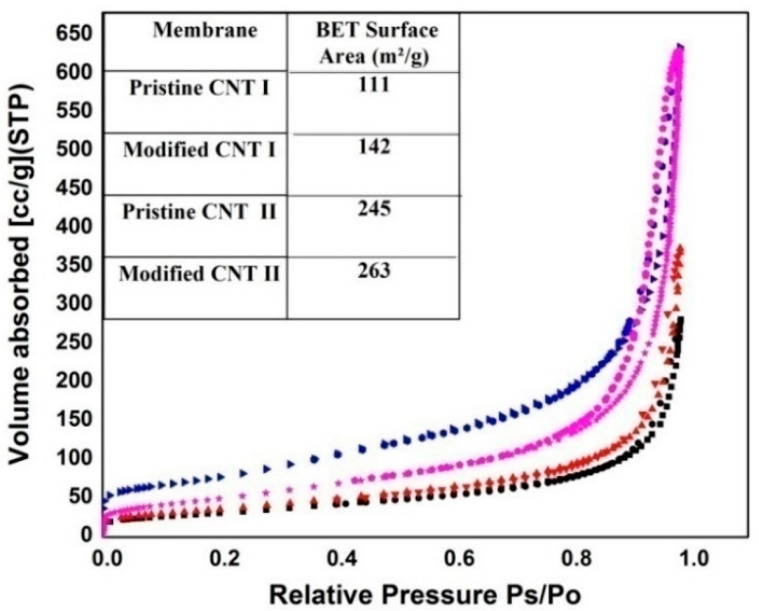
Nitrogen adsorption isotherms and Brunauer–Emmett–Teller (BET) surface area for the pristine and surface modified CNTs.

The specific surface area was measured to describe the effect of CNTs aspect ratio and surface modification on providing more active sites on the CNTs walls. As shown in Figure 4, the aspect ratio has a great effect on increasing the CNTs surface area. In literature, it was proven that larger diameter CNTs can contain higher contents of metal impurities which cause a decrease of the surface area; and longer CNTs can also provide more active sites for N_2_ adsorption than shorter ones [50]. As a result, the surface area for CNT-I was about 245 m^2^/g compared to 111 m^2^/g for CNT-II. Moreover, the surface modification of CNTs had also a clear effect on the surface area. From Figure 4, an increase in the surface area to 263 m^2^/g and 142 m^2^/g for modified CNT-I and CNT-II, respectively, was observed. It was previously discussed that the surface modification increases the specific surface area via opening the tube ends and generating more sidewall defects, which provide extra active sites [51]. Furthermore, functionalization of CNTs disturbs the π–π interaction and van der Waals forces between the tubes, making CNTs debundle and consequently increase the specific surface area [52].

### 3.2. Characteristics of Buckypapers

**Figure 5:**
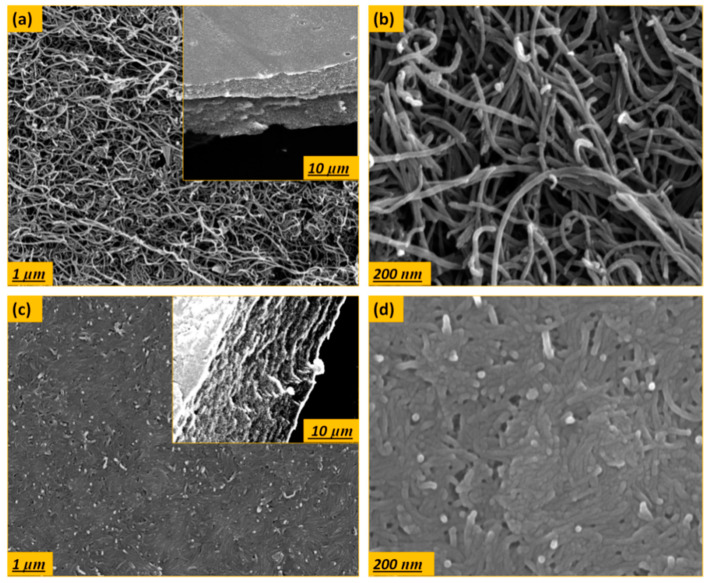
SEM images with low and high magnification for the top surface and cross-section of the (**a**,**b**) pristine and (**c**,**d**) surface modified CNT-I buckypapers.

The surface structure of pristine and modified buckypapers was investigated using SEM. As shown in Figure 5, the buckypapers exhibited homogeneous distribution of CNTs bundles without CNTs aggregates, as a result of good dispersion stability. The pore size distribution of buckypapers was estimated using SEM as well as capillary flow porometry (Table 2). There are two different factors that could affect the buckypaper membranes pore size. Firstly, the CNTs aspect ratio has an influence; it can be found in literature that the higher the CNTs aspect ratio is, the smaller buckypaper pore size is formed [53]. The analysis revealed that the pristine CNT-I buckypapers have an average pore size of 57 and 26 nm, extracted by SEM and flow porometry, respectively. The pristine CNT-II showed pore size of 63 and31 nm, extracted by SEM and flow porometry, respectively. The results confirm that the CNT-I with higher aspect ratio yielded smaller buckypaper pore size compared to CNT-II. Secondly, regarding the effect of surface modification on the pore structure, the SEM images showed more dense structure with closely stacked bundles for the modified buckypapers with average pore size of 27 and 33 nm for modified CNT-I and CNT-II, respectively. The mean pore diameters from flow porometry for both buckypapers were 17 and 22 nm, respectively. The narrower pore size and dense structure of the surface modified buckypapers can be explained by the effect of acid modification on etching the tubs forming narrower pore diameters [54,55,56].

There are two different types of pore structures that can be formed in the CNTs buckypaper membranes. One is the intra-bundle pores that are found in the interior structure of CNTs bundles; this type of pore is expected to be comparable to the CNTs diameters. The other type is the inter-bundle pores, which are formed between the CNTs bundles, and has larger size. The latter type of pore structures reflect the homogeneity of CNTs networks and is more effective for membrane filtration purposes [57,58]. The obtained pore size distribution of the buckypapers of the present study is in close agreement with their ported studies for the MWCNTs buckypapers which are in range of 5–70 nm [15]. Previous study reported that the SEM pore size of pristine MWCNT and MWCNT-COOH buckypapers are 80 and 55 nm [15], respectively. Another study discussed the difference in mean flow pore diameter between compressed and uncompressed buckypapers, i.e., 27 and 33 nm, obtained through capillary flow porometry [59].

### 3.3. Filtration Performance of Buckypapers

#### 3.3.1. Pure Water Permeability

The physical characteristics and pure water permeability of buckypapers are presented in Table 3. The membranes thickness was in range of 150 µm. In terms of membranes surface properties, the pristine buckypapers showed hydrophobic nature with a contact angle of about (140 ± 5°), while for the modified buckypapers, the contact angle was around 40° as a result of successful functionalization. The water permeability for modified buckypapers was noticeably increased compared to the pristine ones, mainly due to introducing oxygen functional groups onto the membrane surface. A similar study introduced the difference in water flux between raw and surface modified CNTs using the same modification method [31]. However, they reported a much lower permeability of 18 and 40 L·m^−2^h^−^1·bar^−1^ for pristine and functionalized buckypapers, respectively. Another study reported the water permeability of membranes from functionalized MWCNT with –NH_2_ and –COOH groups of 13 L·m^−2^h^−1^·bar^−1^and 17 L·m^−2^h^−1^·bar^−1^, respectively [15]. The water permeability through CNTs buckypapers can be affected by the difference in CNTs interior structure and the membrane’s fabrication process such as sonication time, centrifugation, deposition by filtration and drying mechanisms.

#### 3.3.2. Removal of Humic Acid (HA)

**Figure 6:**
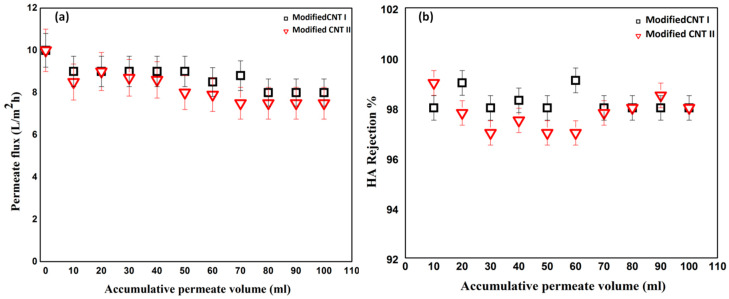
(**a**) Flux and (**b**) rejection for filtration of a solution of humic acid (HA) (5 ppm) through surface modified buckypaper (membrane area 17.35 cm^2^).

**Figure 7:**
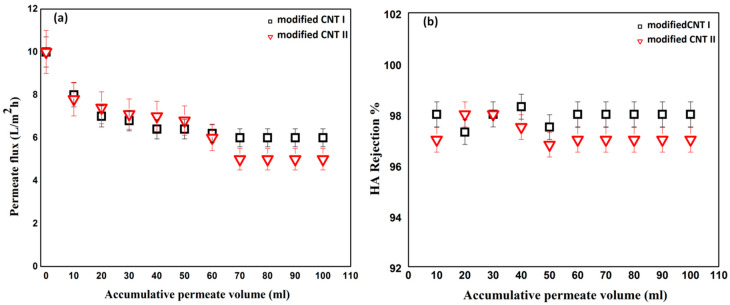
(**a**) Flux and (**b**) rejection during filtration of solution of humic acid (10 ppm) through surface modified buckypapers (membrane area 17.35 cm^2^).

**Figure 8:**
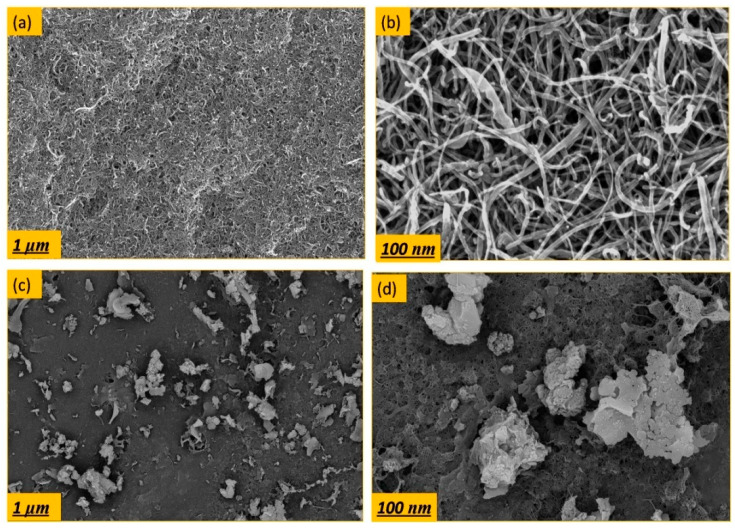
SEM images of low and high magnification surface for the modified buckypapers (**a**,**b**) before and (**c**,**d**) after HA (10 ppm) filtration.

The surface modified buckypapers were tested for the removal of HA from water. Figure 6 and Figure 7 show the effect of surface modification on the HA flux and rejection over 100 mL of accumulated permeate at different concentrations of 5 and 10 ppm. During HA filtration, initial permeate flux was set at 10 L·m^−2^h^−1^ in order to study the flux decline behavior of modified buckypapers. As shown in Figure 6a, the modified buckypaper exhibited excellent antifouling performance for HA with only 20% flux decline at the end of filtration cycle. Similarly, Figure 7a represents the removal of a very high fraction of HA (initial concentration 10 ppm) through the modified membranes. Flux decline of 40% as a result of cake-layer formation onto the membrane surface at higher HA concentration was observed. The surface modified buckypapers showed high rejection efficiency for low and high HA concentrations. As presented in Figure 6b and Figure 7b, the membranes rejection for humic acid was >97%. The antifouling and retention efficiencies of modified buckypapers to humic acid can be attributed to the size exclusion and electrostatic repulsion mechanisms. The relatively small pore size and surface negative charge (Figure S2; Supplementary Materials) make the penetration of high molecular weight fraction of HA into the membrane very much hindered. A layer of HA will be formed on the membrane surface which will largely contribute to the rejection. Figure 8 shows the surface of buckypaper before and after 3 fouling and cleaning cycles. Similar work reported the rejection of modified buckypapers to HA (10 ppm) up to 93%for 180 mL of HA solution over 4.9 cm^2^ membrane area, while the water flux declined to 20% from its initial value after the HA filtration [31]. Another study introduced a comparison between a PVDF membrane and –COOH functionalized CNTs buckypaperfor the removal of HA [60]; the study revealed that the HA flux was reduced to 91% after 6 h of HA filtration through a membrane area of 17.34 cm^2^, while the rejection efficiency maintained at 94%.

#### 3.3.3. Separation of Oil-In-Water Emulsion

**Figure 9:**
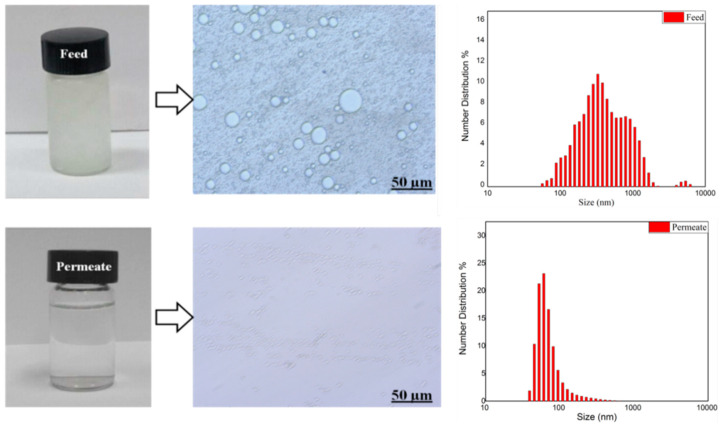
Photographs of feed (Shell oil, 5 wt%), and permeate, accompanied with optical micrograph images and droplet size analysis by dynamic light scattering (DLS) of feed and permeate.

The separation performance of pristine CNTs to oil-in-water (O/W) emulsion was evaluated. Figure 9 shows the size distribution of oil droplets in the feed and permeate combined with photographs and optical micrograph images. As illustrated in Figure 9, a wide distribution of particle size was observed (50–1000 nm) in the feed emulsion, while only oil droplets of about <60 nm in size were observed in the permeate. The particle size analysis confirms the successful separation of large oil droplets, while the presence of oil in permeate could be attributed to the permeation of smaller oil droplets under pressure through the surface and internal pore structure of the membranes which have pore size in the range of 26–57 nm (cf. Table 2).

**Figure 10:**
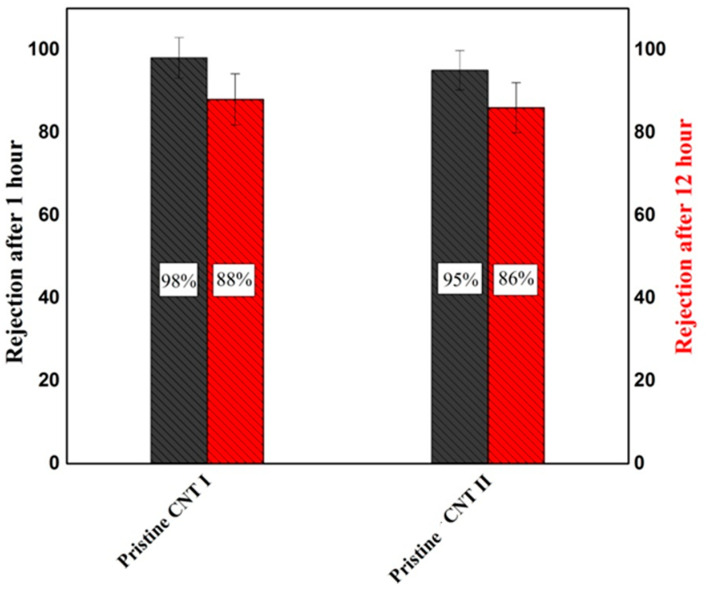
The oil rejection of oil-in-water emulsion for CNT-I and CNT-II buckypapers after 1 h and 12 h of filtration.

Oil-in-water emulsion separation was studied by filtering a total 100 mL of feed emulsion through the membrane. After 1 h, about 5 mL of the feed emulsion was filtered across the active surface area of 17.35 cm^2^, while after 12 h about 70 mL was filtered leaving a layer of oil deposited onto the membrane surface. Figure 10 represents the rejection of pristine CNT-I and CNT-II to O/W emulsion; the figure illustrates that after 1 h, remarkable separation of oil from water of approximately 98% and 95% for pristine CNT-I and CNT-II, respectively, was achieved. After 12 h the rejection declined to 88% and 86%, respectively. During the filtration of oil-in-water emulsion, emulsified oil droplets are deposited onto the membrane surface which partially blocks the membrane pores at the early stage of filtration. With prolonged filtration time, more oil droplets will accumulate or coalesce on the buckypaper surface forming larger oil droplets layer which can contribute to the decline in the separation efficiency [61].

#### 3.3.4. Sorption of Organic Liquids

**Figure 11:**
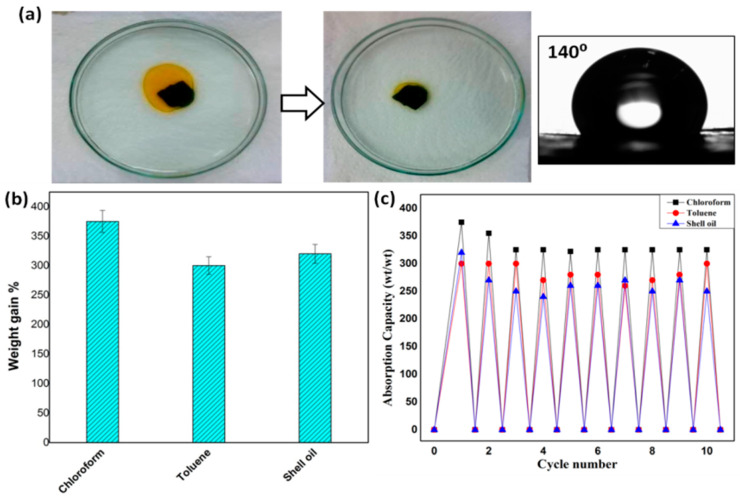
(**a**) Photographs showing the absorption of Shell oil by pristine CNT I buckypaper combined with surface contact angle, (**b**) sorption capacity of CNT I for chloroform, toluene and Shell oil, and (**c**) recyclability of pristine buckypaper over 10 cycles.

The sorption capacities for chloroform, toluene and Shell oil through pristine CNTs buckypapers were evaluated. Pristine CNT-I buckypaper was chosen for the sorption experiments due to its super hydrophobicity and higher efficiency in oil removal under filtration conditions (cf. Section 3.3.3), which makes it a very promising candidate in the removal of oil and various non-polar organic solvents from water. Figure 11a shows the sorption process of Shell oil using pristine CNT-I buckypaper; most of the oil applied onto buckypaper surface was absorbed within a few minutes. The sorption capacity was quantitatively studied by obtaining the membrane weight gain compared to the initial weight at the end of sorption cycle. As shown in Figure 11b, the pristine buckypaper showed high sorption capacity of ~300–350 wt%, which is two times higher than in the reported studies [62]. The recyclability and robustness of the buckypaper were studied by repeating the sorption experiment over 10 cycles. Figure 11c shows insignificant loss in membrane absorption capacity (<10%), which confirms the potential of buckypaper membranes in practical applications.

## 4. Conclusions

Pristine (hydrophobic) and surface modified (hydrophilic) buckypaper membranes, prepared from two different CNTs with different aspect ratios, were utilized for the removal of humic acid from water and the separation of oil-in-water emulsion. In addition, the sorption capacity of hydrophobic buckypapers to several organic solvents was evaluated. The results showed higher permeability compared to previous reports of similar buckypapers and low flux decline for modified buckypapers with high HA rejection performance (>97%) for 100 mL of permeate over 17.35 cm^2^ membrane area via size exclusion and electrostatic repulsion mechanisms. The pristine buckypaper obtained efficient oil-in-water emulsion separation through accumulating the oil droplets onto the external and internal membrane surface. Moreover, the sorption experiment of pristine buckypapers revealed significant absorption capacity for 10 sorption cycles. The results of the study emphasizes the large potential of CNTs buckypaper membrane for environmental applications.

## Figures and Tables

**Table 1:** Chemical vapor deposition (CVD) parameters used for the preparation of the two different carbon nanotubes (CNTs).

Parameter	CNT-I	CNT-II
Catalyst molar ratio	6:0.1:18	1:0.1:13
Active gas	Methane (CH_4_)	Acetylene (C_2_H_2_)
Reaction temperature	1000 °C	700 °C
Reaction time	10 min	30 min
Carrier gas flow rate	300 mL/min	900 mL/min
Active gas flow rate	75 mL/min	100 mL/min

**Table 2:** Average pore size of buckypapers extracted from the SEM and capillary flow porometry analysis.

Sample	SEM Pore Size (nm)	Mean Flow Pore, MFP (nm)
Pristine CNT-I	57 ± 3	26
Modified CNT-I	27 ± 2	17
Pristine CNT-II	63 ± 5	31
Modified CNT-II	33 ± 5	22

**Table 3:** Characteristics of buckypapers.

Property	Pristine CNT-I	Modified CNT-I	Pristine CNT-II	Modified CNT-II
Thickness (µm)	153 ± 3	158 ± 5	150 ± 10	150 ± 4
Contact angle (°)	135 ± 7	45 ± 2	140 ± 5	42 ± 9
PWP (L·m^−2^h^−1^·bar^−1^)	80 ± 6	172 ± 4	88 ± 2	196 ± 8

## Supplementary Materials

The following are available online at https://www.mdpi.com/2077-0375/10/5/97/s1, Figure S1: The effect of dispersion stability on buckypaper formation, Figure S2: The particle size of pristine and surface modified CNTs dispersions in NMP and aqueous solvent without dilution, inserted with the zeta potential of CNTs dispersions at pH 7, Figure S3: (a) BJH pore size distribution inserted with buckypapers membranes surface area and pore volume, (b) nitrogen adsorption-desorption isotherms of buckypapers, Figure S4: SEM images combined with threshold segmentation images for pristine (a,b), and surface modified (c,d) CNT-II, respectively.
