# Supplementary material for: Tailored CNTs Buckypaper Membranes for the Removal of Humic Acid and Separation of Oil-In-Water Emulsions
Source: Membranes (Basel). 2020 May 12;10(5):97. doi: 10.3390/membranes10050097 (PMC7281685; doi:10.3390/membranes10050097)
Supplement: Supplementary file 1 [file membranes-10-00097-s001.pdf]

membranes

Supplementary Materials

Tailored CNTs Buckypaper Membranes for the Removal of Humic Acid and Separation of Oil-in-Water Emulsions

Eman Elnabawy [1], Ibrahim M.A. Elsherbiny [2], Ahmed M. A. Abdelsamad [2,3], Badawi Anis [4], Abdelwahab Hassan [1], Mathias Ulbricht [2] and Ahmed S. G. Khalil [1,5,*]

1 Physics Department and Center for Environmental and Smart Technology, Faculty of Science, Fayoum University, 63514 Fayoum, Egypt; eman.elnabawy@smartci.alexu.edu.eg (E.E.); aha08@fayoum.edu.eg (A.H.)
2 Lehrstuhlfür Technische Chemie II, and Center for Water and Environmental Research (ZWU), University of Duisburg-Essen, 45141 Essen, Germany; ibrahim.elsherbiny@uni-due.de (I.M.A.E.); ahmed.abdelsamad@stud.uni-due.de (A.M.A.A.); mathias.ulbricht@uni-due.de (M.U.)
3 Water Pollution Dept, National Research Centre, 33 El-Bohouth St., Dokki, Giza 12622, Egypt
4 Spectroscopy Dept, Physics Division, National Research Centre, 33 El-Bohouth St., Dokki, Giza 12622, Egypt; badawi.ali@gmail.com
5 Materials Science & Engineering Department, School of Innovative Design Engineering, Egypt-Japan University of Science and Technology (E-JUST), 179 New Borg El-Arab City, 21934 Alexandria, Egypt
* Correspondence: asg05@fayoum.edu.eg

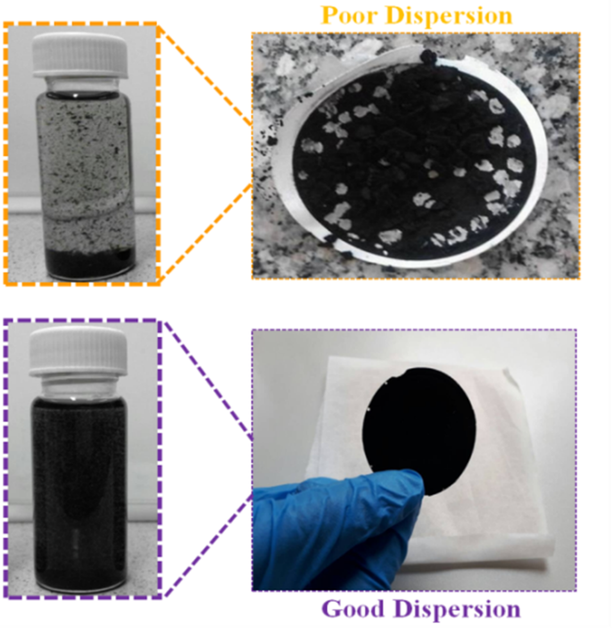

Figure S1. The effect of dispersion stability on buckypaper formation.

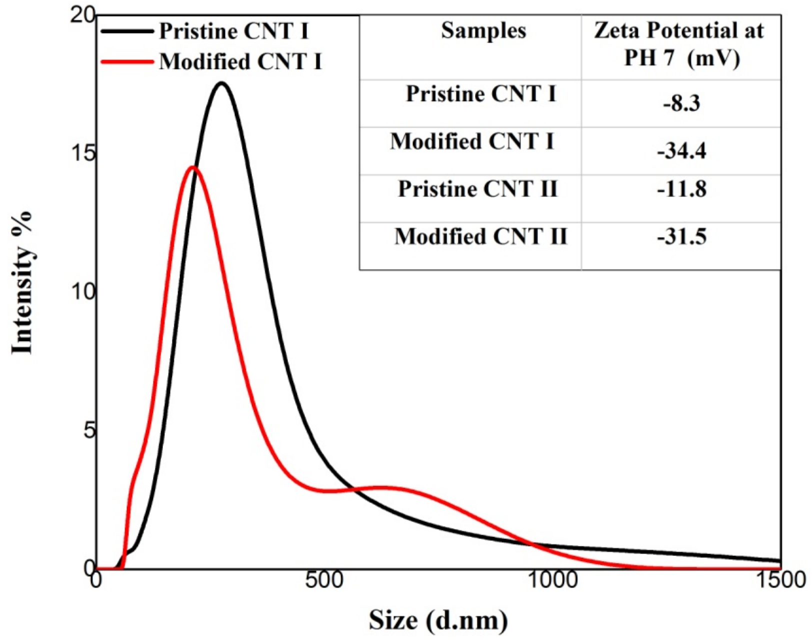

Figure S2. The particle size of pristine and surface modified CNTs dispersions in NMP and aqueous solvent without dilution, inserted with the zeta potential of CNTs dispersions at PH 7.

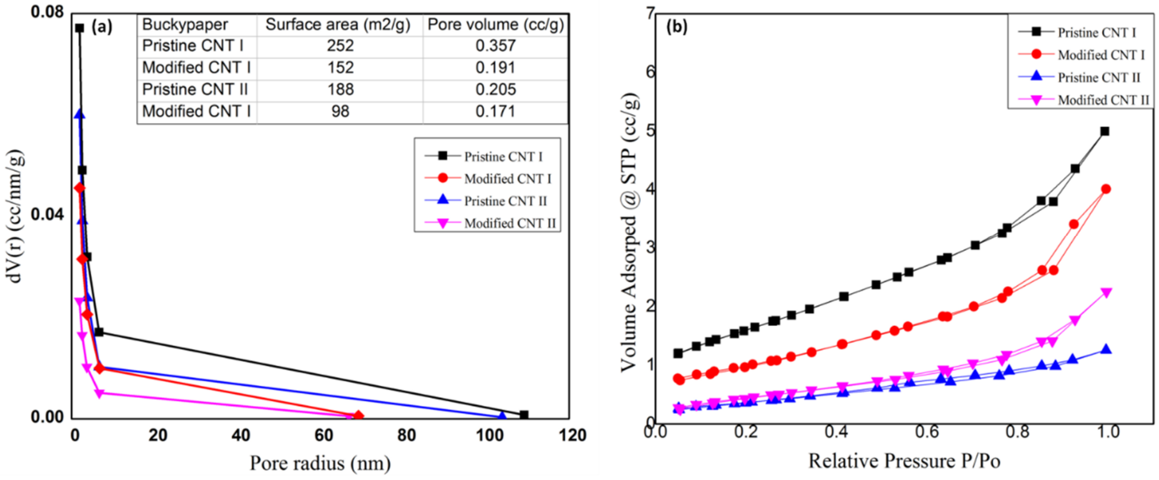

Figure S3. (**a**) BJH pore size distribution inerted with buckypapers membranes surface area and pore volume, (**b**) The N_2 adsorption-desorption isotherm of buckypapers.

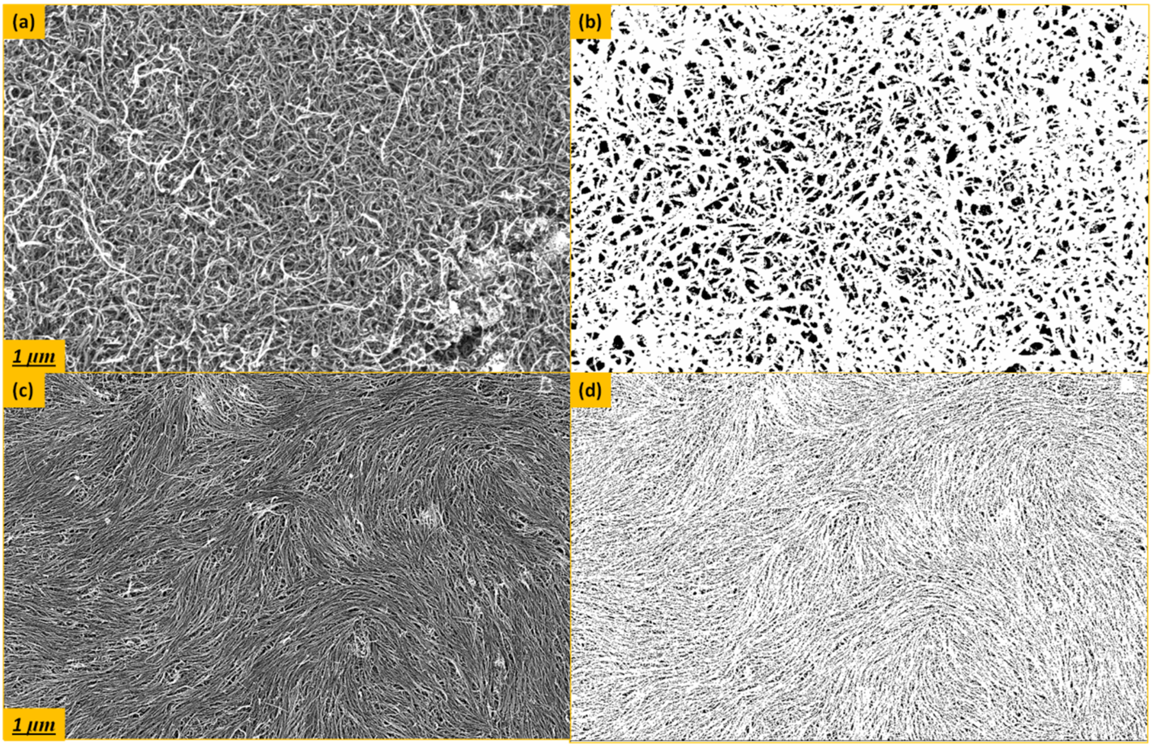

Figure S4. SEM images combined with threshold segmentation images for pristine (**a**,**b**), and surface modified (**c**,**d**) CNT-II, respectively.
